# Editorial: Advances in diagnostics and management of adrenal tumors

**DOI:** 10.3389/fendo.2025.1543773

**Published:** 2025-01-30

**Authors:** Piotr Glinicki, Nadia Sawicka-Gutaj, Marta Araujo-Castro

**Affiliations:** ^1^ EndoLab Laboratory, Centre of Postgraduate Medical Education, Warsaw, Poland; ^2^ Department of Endocrinology, Centre of Postgraduate Medical Education, Warsaw, Poland; ^3^ Department of Endocrinology, Metabolism and Internal Medicine, Poznań University of Medical Sciences, Poznań, Poland; ^4^ Endocrinology & Nutrition Department, Hospital Universitario Ramón y Cajal, Madrid, Spain; ^5^ Instituto de Investigación Biomédica Ramón y Cajal (IRYCIS), Madrid, Spain

**Keywords:** adrenal tumors, pheochromocytoma, paraganglioma, adrenocortical carcinoma, Cushing syndrome, primary aldosteronism, biomarkers

Adrenal tumors are a heterogeneous group of tumors characterized by diverse biology and clinical courses. They may be associated with high malignant potential and/or hormonal activity, leading to multiple complications, with cardiovascular issues being the most prevalent. Diagnosing adrenal tumors often requires complex and costly laboratory and imaging procedures, which, unfortunately, in many cases, do not provide complete certainty about the diagnosis or the nature of the neoplastic process (benign, malignant, or hormonally active). A major challenge is that hormonally active adrenal tumors or potentially malignant adrenal lesions are rare or very rare. As a result, conducting research on them is particularly difficult. One of the current research directions for this group of neoplasms is biochemical research in metabolomics and proteomics, supported by bioinformatics techniques based on artificial intelligence (AI).

## The latest in biochemical diagnostics of adrenal tumors

New biochemical methods for the diagnosis and monitoring of patients with adrenal tumors are currently being developed in two parallel directions. The first line of research involves classical immunological (immunochemical) methods based on the antigen-antibody reaction. In particular, automated immunochemical methods based on the chemiluminescence reaction (CLIA, ECLIA) are being evaluated, primarily as widely available, inexpensive, and easy-to-use screening tools (1). These studies include the use of these methods as screening tests in the diagnosis of patients with suspected primary aldosteronism (PA), the feasibility and clinical value of assaying various hormones, e.g. aldosterone, direct renin concentration (DRC), plasma renin activity (PRA), and cortisol in tests aimed at differentiating between different subtypes of PA, and, thirdly, the feasibility of assaying free hormone fractions in various biological materials (e.g., free cortisol in saliva) (2–4). The other direction involves chromatographic methods. New possibilities for biochemical diagnostics are opened by metabolomics research using modern analytical techniques: Liquid chromatography–mass spectrometry (LC-MS/MS), Gas chromatography/mass spectrometry (GC/MS) and others. The development of various chromatographic techniques, especially the LC-MS/MS technique, makes it possible to determine multiple hormones during a single analysis (e.g., steroid hormone profile in plasma or daily urine collection, free metanephrine profile in plasma, etc.) in routine diagnostics (5, 6). The introduction of the ability to determine hormone panels using various chromatographic techniques (mainly LC-MS/MS and GC/MS) into biochemical diagnostics enables new studies both in screening diagnosis of adrenal tumor lesions and in other diagnostic and therapeutic areas. An additional unique feature of hormonal profile studies in biological material from patients with adrenal tumors is their personalized nature, which can be used in the individual diagnostic-therapeutic process in a given group of patients (7, 8).

## The latest in molecular diagnostics of adrenal tumors

Progress in the field of molecular studies in adrenal tumors has been made in the molecular characterization of pheochromocytoma, adrenal Cushing’s syndrome, PA, and adrenocortical carcinoma (ACC). F or pheochromocytomas, although some older studies reported a prevalence of germline genetic variants in 10% - 15% of the patients, more recent studies performed with NGS (Next-generation sequencing) technology describe genetic variants in up to 35% - 40% of patients (9–11). The prevalence of genetic variants in these patients has been increasing over the years as new genes such as *CSDE1, H3F3A, MET, MERTK* and *IRP1*, have been discovered (10). Furthermore, recent advances in the study of pheochromocytomas have revealed new molecular events in these tumors. In the context of adrenal Cushing’s syndrome, one of the most recent discoveries has taken place in patients with primary bilateral macronodular adrenal hyperplasia (PBMAH): the discovery of the role of variants in *KDM1A* in GIP-mediated cases of Cushing’s syndrome. It was reported that 100% of the patients with PBMAH and GIP-responsive Cushing’s syndrome had a germline variant in *KDM1A*, compared with 0% of patients from the control group (11). The discovery of genetic alterations such as *ARMC5* and *KDM1A* in PBMAH allows early detection of PBMAH in patients’ relatives (12). Regarding PA, several genetic defects in the germline or somatic state have been identified. Although only 5% of PA are familial (13), it is currently known that approximately 90% of aldosterone-producing adenomas (APAs) are due to somatic variants in genes encoding ion channels or transporters including *KCNJ5, CACNA1D, ATP1A1*, and *ATP2B3* (14). In recent years, new somatic variants have been identified, including a new one in *CACNA1H* (15). In addition, more recently, the co-existence of *CTNNB1* with *G Protein Subunit Alpha Q (GNAQ)/G Protein Subunit Alpha 11 (GNA11)* variants has been documented in 59% of APAs (16). Finally, advances in the genetics of ACC have also been reported. Although there are not many current therapeutic options directly targeting reported ACC alterations, some studies have detected variants in *TP53, BRD9, TERT, CTNNB1, CDK4, FLT4* and *MDM2* as potentially targetable genetic alterations in patients with ACC (17). Utilizing blood-based NGS to characterize genomic alterations in advanced ACC is feasible in over 80% of patients, with 50% of them being potentially targetable (18). Thus, in conclusion, advances in the knowledge of the genetic context of functioning adrenal tumors have allowed a better characterization of these tumors, with important implications in the management of these patients, including the personalization of follow-up and treatment, and its importance in the face of genetic counseling for patients and their relatives.

The current Research Topic remains of high scientific and clinical interest and includes 23 articles.

The aim of the first article (Araujo-Castro et al.) was to compare the clinical presentation and laboratory hormonal diagnostics in patients with two forms of PA: familial hyperaldosteronism (FH) and primary hyperaldosteronism (PA). The study was a meta-analysis based on a systematic review of the literature to identify patients with FH. A total of 360 FH cases (246 FH type I, 73 type II, 29 type III and 12 type IV) and 830 sporadic PA patients (from the SPAIN-ALDO registry) were included in the study. Analysis of the results showed a different clinical presentation in patients with FH-I and III compared to sporadic forms of PA. In this regard, FH-I is characterized by a low prevalence of hypokalemia, while FH-III is characterized by severe aldosterone over-secretion causing hypokalemia in more than 85% of patients. The clinical and hormonal phenotype of types II and IV is similar to that of patients with sporadic PA.

Another original article (Liu et al.) focused on the evaluation of postoperative management of patients with pheochromocytoma assessing hemodynamic stability as one of the main causes of serious complications after surgical treatment, and in extreme cases leading to patient death.

The aim of this retrospective study by Canu et al. was to evaluate changes in Luteinizing hormone (LH), sex hormone binding globulin (SHBG), total testosterone (TT) and calculated free testosterone (cFT), the prevalence and type of hypogonadism and sexual function, the latter before and after androgen replacement therapy (ART) in patients with ACC treated with adjuvant mitotane therapy (AMT). The authors suggested monitoring LH, SHBG, TT and cFT and sexual function during AMT, and starting ART in hypogonadal patients with ACC with sexual dysfunction.

The research group of Araujo-Castro et al. sought to evaluate the prevalence of recurrence in patients with pheochromocytomas and sympathetic paragangliomas (PGLs; collectively referred to as PPGLs) and to identify predictors of recurrence (local recurrence and/or metastatic disease). This retrospective multicenter study included information on 303 patients with PPGLs in follow-up in 19 Spanish tertiary hospitals. The conclusions of this study are that since PPGL recurrence can occur at any time after the initial diagnosis of PPGL, it is recommended to closely follow up with all patients with PPGL, especially those with a higher risk of recurrence.

In a different article, researchers (Vetrivel et al.) performed a transcriptomic analysis of adrenal signaling pathways in different forms of endogenous Cushing’s syndrome to define areas of dysregulation and targets that can be treated. NGS analysis was performed on adrenal samples from patients with PBMAH (n = 10) and control adrenal samples (n = 8). Validation groups included cortisol-producing adenoma (CPA, n=9) and samples from patients undergoing bilateral adrenalectomy for Cushing’s syndrome (BADX-CD, n=8). This project concluded that the therapeutic effect was independent of the actions of ACTH, postulating a promising application of PPARG activation in endogenous hypercortisolism.

According to Díaz-López et al., severe hypokalemia leading to rhabdomyolysis (RML) in PA is a rare occurrence, with only a few cases reported in the last four decades. Their systematic review and case report aimed to gather all published data regarding hypokalemic RML as a presentation of PA, in order to contribute to the early diagnosis of this extremely rare condition. Early detection and management are essential to reduce the frequency of complications such as acute kidney injury.

Mansour et al. investigated an integrated diagnostic approach to predict the source of aldosterone overproduction in PA. A total of 269 patients with PA from the prospective German Conn Registry were included in this study. The integration of clinical parameters into a radiomics machine learning model improved the prediction of the source of aldosterone overproduction and subtyping in the patients.

Szatko et al. prepared a mini-review summarizing current data on the pathophysiological pathways of cardiac damage caused by catecholamines, the clinical presentation of PPGL-induced cardiomyopathies, and discussion of treatment options.

The aim of another included study (Berndt et al.) was to assess the diagnostic value of salivary cortisol and cortisone in patients with suspected hypercortisolism including 155 patients with adrenal incidentaloma, and 54 patients with suspected Cushing’s syndrome. The authors concluded that late-night salivary cortisol is not sufficiently sensitive or specific to be used for screening patients with suspected hypercortisolism. Instead, late-night salivary cortisone appears to be a promising alternative in patients with adrenal incidentaloma and salivary cortisone at 8 a.m. following the dexamethasone suppression test in patients with suspected Cushing’s syndrome.

The research group of Choromańska et al. assessed the total antioxidant/oxidant status in the plasma and urine of patients with adrenal tumors. The study group consisted of 60 patients (31 women and 29 men) with adrenal masses, classified into three subgroups: non-functional incidentaloma, pheochromocytoma and Cushing / Conn adenoma. The authors analyzed various biomarkers of antioxidant activity: Total Antioxidant Capacity, Total OxidantStatus, Oxidative Stress Index and Antiradical Activity (Radical-Scavenging Activity Assay, Ferric-Reducing Antioxidant Power). Both plasma and urine redox biomarkers can be used to assess systemic antioxidant status in patients with adrenal tumors.

Sun et al. developed a computed tomography (CT) -based radiological-clinical prediction model for evaluating the surgical difficulty of treatment using RPLA (Retroperitoneal Laparoscopic Adrenalectomy) based on data from 398 patients with adrenal tumors. The authors developed a radiological-clinical prediction model to predict the difficulty of RPLA procedures. This model was suitable, accessible, and helpful for individualized surgical preparation and reduced operative risk.

A meta-analysis by Li et al. focused on comparing the advantages of robotic posterior retroperitoneal adrenalectomy (RPRA) over laparoscopic posterior retroperitoneal adrenalectomy (LPRA). A total of 675 patients were included. It was found that RPRA is associated with a significantly shorter hospital stay compared to LPRA, while showing a comparable operative time, blood loss, conversion rate, and complication rate.

The following article (Sun et al.) described machine learning models for predicting the difficulty of retroperitoneal laparoscopic adrenalectomy by combining clinical and radiomic characteristics. These models can help surgeons evaluate surgical difficulty, reduce risks, and improve patient benefits.

In another study (Urusova et al.), the authors introduced a universal mathematical model for the differential diagnosis of all morphological types of ACC in adults. The method involves determining eight diagnostically significant indicators that enable the calculation of the probability of ACC development using specified formulas.

Zhanghuang et al. presented an interesting case report of a bilateral adrenal giant medullary lipoma and performed a review of the literature. Patients with adrenal myelolipoma complicated with sexual development disorders can be monitored after resection of the myelolipoma, prior to oculoplastic surgery. In some cases, patients with sexual development disorders may experience spontaneous relief of abnormal manifestations of the external genitalia.

Another article (Deng et al.) presented a case of ACC with liver metastases treated with systemic antitumor therapy combined with local therapy for liver lesions (mitotane combined with TACE+MWA). The treatment outcome was a partial response, and the progression free survival of the patient has been extended to approximately 28 months so far, with survival when the study was completed (September 2022).

The Research Group of Enguita et al. found significant differences in the miRNA expression profiles of paragangliomas and pancreatic neuroendocrine tumors, leading to the identification of 6 key miRNAs (miR-10b-3p, miR-10b-5p, and the miRNA families miR-200c/141 and miR-194/192) that can effectively differentiate between the two types of tumors.

A group of authors from Italy (Delbarba et al.) included a total of 24 patients with ACC in their study. Testosterone deficiency was reported in 10 patients (41.7%) at baseline. It was found that mitotane therapy exposes these patients to a further elevated risk of hypogonadism, which should be promptly recognized and treated as it may have a negative impact on quality of life.

Kimura et al. presented a prospective study on mixed corticomedullary tumors of the adrenal gland, which are extremely rare tumors characterized by an admixture of steroidogenic cells and chromaffin cells in a single tumor mass simultaneously producing adrenocortical hormones and catecholamines; in some cases, it is associated with ectopic adrenocorticotropic hormone.

An included review (Constantinescu et al.) described clinically “silent” PPGLs which are characterized by the absence of signs and symptoms associated with catecholamine excess. “Non-secretory” tumors are those with an absence of clear catecholamine secretory activity, “biochemically negative” PPGLs are those characterized by plasma or urinary metanephrines below the upper cut-offs of reference intervals and “non-functional” tumors are those with no catecholamine synthesis as determined by measurements of catecholamines in the tumor tissue.

Another case report (Weng et al.) highlighted the remarkable response of a patient with an ACC microsatellite instability-high tumor, *MLH1* splice variant, and high tumor mutational burden to treatment with a novel combination of mitotane, etoposide, paraplatin and sintilimab.

The research group of Zhang et al. investigated steroid profiling by LC-MS/MS led us to select DHEA as a candidate reference hormone for cortisol secretion. Lateralization and different steroid ratios showed that each steroid and all three steroidogenic pathways may be affected in patients with PBMAH. In patients with germline *ARMC5* variants, the androgen pathway was particularly dysregulated.

In the final study to be included (Mellid et al.) 23 patients carrying germline *NF1* variants were found to have additional pathogenic germline variants in *DLST* (n=1) and *MDH2* (n=2), and two somatic variants in *H3-3A and PRKAR1A*, revealed by targeted sequencing. Thus, the authors concluded that variants affecting genes involved in different pathways (pseudohypoxic and receptor tyrosine kinase signaling) co-occurring in the same patient could provide a selective advantage for the development of PPGL and explain the variable expressivity and incomplete penetrance observed in some patients.

In summary, this Research Topic illustrates the challenges in the diagnosis and treatment of patients with adrenal tumors along with new diagnostic and therapeutic options.
